# Increasing habitat complexity on seawalls: Investigating large‐ and small‐scale effects on fish assemblages

**DOI:** 10.1002/ece3.3475

**Published:** 2017-10-14

**Authors:** Rebecca L. Morris, M. Gee Chapman, Louise B. Firth, Ross A. Coleman

**Affiliations:** ^1^ School of Life and Environmental Sciences Centre for Research on Ecological Impacts of Coastal Cities The University of Sydney Sydney NSW Australia; ^2^ School of Biological and Marine Sciences Plymouth University Plymouth UK

**Keywords:** artificial habitat, camera, eco‐engineering, infrastructure, retrofitting biodiversity, rock pool, urbanization

## Abstract

The construction of artificial structures in the marine environment is increasing globally. Eco‐engineering aims to mitigate the negative ecological impacts of built infrastructure through designing structures to be multifunctional, benefiting both humans and nature. To date, the focus of eco‐engineering has largely been on benefits for benthic invertebrates and algae. Here, the potential effect of eco‐engineered habitats designed for benthic species on fish was investigated. Eco‐engineered habitats (“flowerpots”) were added to an intertidal seawall in Sydney Harbour, Australia. Responses of fish assemblages to the added habitats were quantified at two spatial scales; large (among seawalls) and small (within a seawall). Data were collected during high tide using cameras attached to the seawall to observe pelagic and benthic fish. At the larger spatial scale, herbivores, planktivores, and invertebrate predators were generally more abundant at the seawall with the added flowerpots, although results were temporally variable. At the smaller spatial scale, certain benthic species were more abundant around flowerpots than at the adjacent control areas of seawall, although there was no general pattern of differences in species density and trophic group abundance of pelagic fish between areas of the seawall with or without added habitats. Although we did not find consistent, statistically significant findings throughout our study, the field of research to improve fish habitat within human‐use constraints is promising and important, although it is in its early stages (it is experimental and requires a lot of trial and error). To advance this field, it is important to document when effects were detected, and when they were not, so that others can refine the designs or scale of habitat enhancements or their study approaches (e.g., sampling protocols).

## 1 INTRODUCTION

Climate change and urbanization are simultaneously degrading coastal ecosystems (Airoldi & Beck, 2007). As the global extent of coastal cities expands and sea levels rise, there will be greater pressure to use coastal infrastructure to protect human assets (Dugan, Airoldi, Chapman, Walker, & Schlacher, 2011). There is increasing concern about the effect of these artificial structures on the marine environment (Airoldi et al., 2005; Bishop et al., 2016; Bulleri & Chapman, 2010). A growing body of research is showing that these artificial structures support different assemblages of organisms to those living on natural rocky substrata and they cannot therefore be considered surrogates for natural habitats (e.g., Chapman, 2003; Firth et al., 2013; Munsch, Cordell, & Toft, 2015b). Regardless, modern societies need infrastructure, and so the balance between maintaining a requirement for infrastructure with the need to sustain natural biodiversity and ecosystem functioning is a current and future challenge (Chapman, Underwood, & Browne, 2017; Dafforn et al., 2015; Firth, Knights et al., 2016).

Planned artificial reefs are often deployed as a tool to enhance commercial fisheries, provide recreational sites for diving, and mitigate anthropogenic impacts to natural reefs (reviewed in Baine, 2001; Feary, Burt, & Bartholomew, 2011). Seawalls and breakwaters have the potential to function as artificial reefs if fish and other organisms respond to the structures in a similar way (Feary et al., 2011). There are many studies describing the diverse fish assemblages of artificial structures (e.g., Clynick, 2008; Pradella, Fowler, Booth, & Macreadie, 2014; Rilov & Benayahu, 1998). The species richness and abundance of fish associated with artificial structures, as opposed to purpose‐built reefs, have been shown to be comparable to that in natural habitats in some studies (Burt, Feary, Cavalcante, Bauman, & Usseglio, 2013; Wen, Pratchett, Shao, Kan, & Chan, 2010), but not in others (e.g. Able, Manderson, & Studholme, 1998; Toft, Cordell, Simenstad, & Stamatiou, 2007). Despite this similarity in species richness however, the identity of species within a fish assemblage is often different between marine infrastructure and natural habitats (Burt et al., 2013; Rilov & Benayahu, 2000).

Loss of biotically complex habitat through urbanization has been met with increasing research efforts to mitigate the negative ecological impacts through “ecological or eco‐engineering” (included as part of “reconciliation ecology” in Rosenzweig, 2003). The application of eco‐engineering to marine infrastructure, however, has been relatively recent and predominantly focused on infrastructure designed to defend shorelines against erosion (reviewed in Chapman & Underwood, 2011; Dafforn et al., 2015). In general, artificial coastal defense structures are designed from an engineering perspective for the sole purpose of protection from erosion and flooding. Eco‐engineering attempts to challenge this tradition by redesigning infrastructure to be multifunctional, benefiting both humans and nature. The aim of ecological enhancement of coastal infrastructure has largely been to increase the overall heterogeneity of substrata and the diversity of benthic species that use these structures as habitat (for reviews see, Chapman & Underwood, 2011; Dafforn et al., 2015; Firth, Knights et al., 2016).

In natural habitats, there is generally a positive relationship between the number of species occupying an area and the complexity of habitats in an area (MacArthur & MacArthur, 1961). Biodiversity of fish has been positively correlated with (1) topographic complexity of their habitat (e.g., Tuya, Wernberg, & Thomsen, 2009) and (2) benthic biodiversity (e.g., Komyakova, Munday, & Jones, 2013), with strong links between the two (Gratwicke & Speight, 2005). Similarly, the species richness and abundance of fish had been shown to be greater around marine infrastructure that has more topographical complexity and a greater cover of complex epibiota than around more simple structures (Clynick, Chapman, & Underwood, 2007; Rilov & Benayahu, 1998).

Sydney is one of a few global hotspots for research on eco‐engineering (Chapman & Underwood, 2011; Strain, Olabarria et al., 2017). One method that was trialed in Sydney that was particularly successful and received significant media attention was the attachment of modified flower pots to vertical seawalls with the aim of increasing diversity of benthic species (Browne & Chapman, 2011, 2014; Morris, unpublished data). Increased benthic diversity and structural complexity provided by eco‐engineered habitats have also been shown to influence fish assemblages in adjacent waters (Sella & Perkol‐Finkel, 2015; Toft, Ogston, Heerhartz, Cordell, & Flemer, 2013), which provides some evidence that eco‐engineering for benthic biota may enhance fish assemblages. This has not, however, been previously evaluated for structures such as flowerpots attached to walls in urbanized harbors.

These flowerpots were originally designed and deployed to add habitat for benthic species living on seawalls, not to change fish abundances. They may, however, have an inadvertent effect on fish, which may then, in turn, affect algae and invertebrates through consumptive and nonconsumptive effects (Connell & Anderson, 1999; Ferrario, Iveša, Jaklin, Perkol‐Finkel, & Airoldi, 2015; Kennelly, 1991). This could enhance or counter the original aim of the flowerpots due to knock‐on effects (i.e., by affecting higher trophic levels, which in turn affects the benthos). Few studies have, however, quantified the effect of eco‐engineering marine infrastructure on fish (but see Toft et al., 2013; Sella & Perkol‐Finkel, 2015; Strain, Morris et al., 2017). Whilst the size of the pots and spatial scales over which they have been deployed were not designed to assess effects on fish, it is important to understand how all components of the ecosystem respond, including fish, so that the primary objective (i.e., to increase biodiversity of seawalls) can be better understood. Further, if the flowerpots at this scale do not have an effect on fish, this is an important result to report. Much of the current literature on eco‐engineering reports on success, as it is harder to report failure (but see, Firth, Browne, Knights, Hawkins, & Nash, 2016). Much can be learnt, however, from trials that do not work in the way planned. It is thus equally, if not even more important to publish negative results as well as successes to inform future eco‐engineering projects (Chapman et al., 2017; Firth, Browne et al., 2016).

Pelagic fish in open water adjacent to artificial structures may be predicted to respond to complexity at a larger spatial scale than do benthic fish (e.g., blennies), which might respond to specific smaller‐scale (cm) structural features (Chapman & Clynick, 2006). For example, small‐bodied fish were found associated with structures present in marinas, whereas larger species moved between structures and the surrounding open water (Clynick, 2008). In addition to a species‐level response, the influence of structural complexity on fish can depend on the trophic group. For example, herbivorous fish were more abundant at more structurally complex reef habitat than in structurally simple lagoon habitats (Vergés, Vanderklift, Doropoulos, & Hyndes, 2011). In contrast, some complex habitats may increase predation risk, and a negative relationship between complexity and fish abundance can be seen at some spatial scales, but not others (Rilov, Figueira, Lyman, & Crowder, 2007). The scale at which organisms respond to different structures is an important consideration in eco‐engineering research which has been largely neglected to date (but see Loke, Ladle, Bouma, & Todd, 2015).

Here, we investigated whether the installation of novel habitats (“flowerpots”) to seawalls in order to provide additional habitat for intertidal benthic species had an effect on the fish assemblage in the waters adjacent to the wall. This was measured at two spatial scales: (1) a large scale (>100 m) compared seawalls with or without flowerpots (among seawalls) and (2) a small scale (1–10 m) compared patches of the wall with or without flowerpots (within a seawall). Fish that were found in open water adjacent to structures (hereafter pelagic) were measured separately to those that were closely associated with the substratum (e.g., gobies and blennies, hereafter benthic). Specifically, it was predicted that (1) the number of species and abundance of different trophic groups of pelagic fish would be greater at the seawall with flowerpots than at control seawalls without those habitats. At the smaller scale, we predicted that (2) the number of species and abundance of different trophic groups of pelagic and (3) all benthic fish would be greater around patches of seawall with flowerpots in comparison with adjacent control areas of the same seawall without flowerpots.

## 2 MATERIALS AND METHODS

### 2.1 Large‐ and small‐scale experimental setup

**Figure 1:**
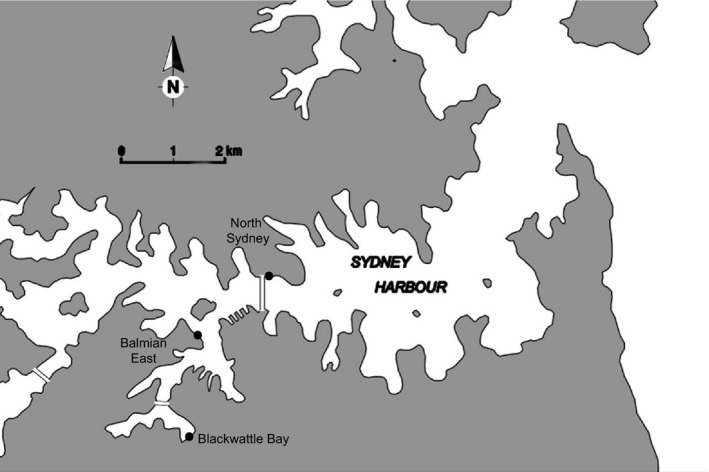
Flowerpots were installed at Blackwattle Bay, Sydney Harbour. Control locations were Balmain East and North Sydney. Double lines across the harbor indicate bridges

**Figure 2:**
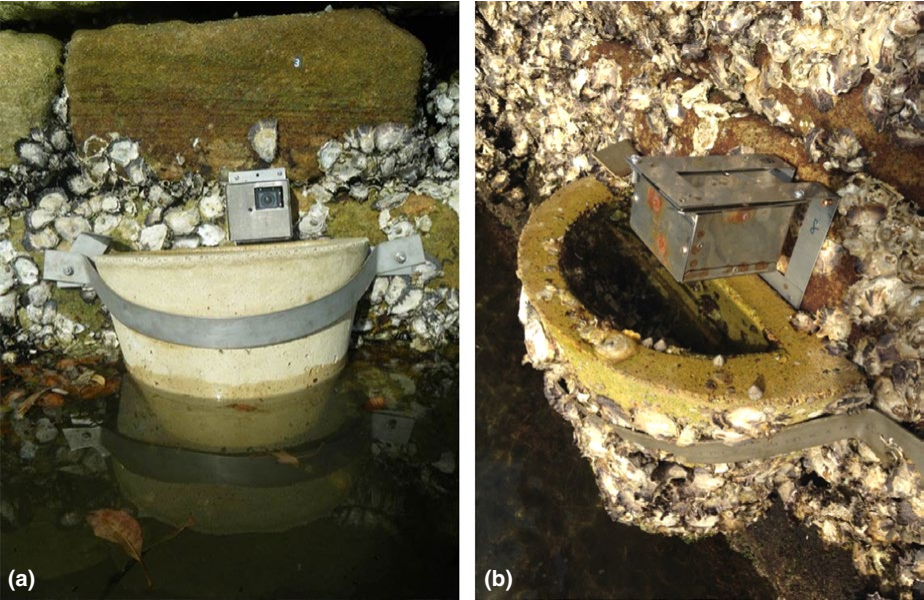
Flowerpot attached to the seawall in Sydney Harbour, Australia with GoPro^®^ camera in (a) stainless steel housing and (b) on L‐shaped bracket

One experimental and two control locations were selected in Sydney Harbour, Australia. The experimental location, which had flowerpots, was at Blackwattle Bay (33.87°S 151.19°E), and the control locations, which had no flowerpots, were at Balmain East (33.51°S 151.11°E) and North Sydney (33.50°S 151.12°E) (Figure 1). Concrete flowerpots (7 L, 315 mm diameter) were fixed to the seawall with a stainless steel bracket (Figure 2), modified from those developed by Browne and Chapman (2011). Ten flowerpots were attached at the mid‐shore tidal level to a sandstone seawall at Blackwattle Bay in February 2014. They were submerged during high tide and retained water during low tide. Two ~20‐m sites were chosen on the seawall, separated by more than 100 m, and five pots were deployed at each site; individual pots were approximately 4 m apart. The location, sites, and number of pots were determined by the local management authority responsible for the seawall. Thus, it was not possible to have multiple seawalls in different locations with flowerpots in this study.

Fish assemblages were sampled using GoPro^®^ cameras (two per site) attached to the seawall in stainless steel housing facing outward (Figure 2a). To test the small‐scale effects (defined as within a seawall) of the flowerpots, in each site at Blackwattle Bay, one camera was deployed above a randomly chosen flowerpot, so that the lip of the flowerpot was in the camera's field of view. One camera was attached to the adjacent seawall without added flowerpots as a control, at least 10 m away. The site and camera setup were repeated in North Sydney and Balmain East to test large‐scale effects (defined as among seawalls). The two cameras in each site were attached to the seawall at approximately the same distances apart as Blackwattle Bay, without flowerpots present.

The cameras were set to take photographs on time lapse every 2 s using the GoPro^®^ setting “5 MP, wide,” and recorded images for approximately 3 hr during high tide. The cameras were switched on ~100 min before high tide; when the mid‐tidal level of the seawall was immersed. The fish assemblage was sampled monthly from March 2014 to February 2015 at each location, and all locations were sampled within the same week every month. The cameras were not moved between sampling times, so recorded the same pots and sections of the wall. This minimized damage to the heritage‐listed seawall.

### 2.2 Data collection and analysis

Due to localized ecological processes, it is highly likely that samples close in time or space may show temporal or spatial dependence (Brown, 1984; Pielou, 1984). Here, because fish were continuously photographed and control and flowerpot treatments were in relatively close proximity, serial autocorrelation was used to determine spatial and temporal independence of replicate video shots (Carlile, Skalski, Batker, Thomas, & Cullinan, 1989; Favaro & Moore, 2015).

Temporal autocorrelation analysis was used to test whether 10‐min time intervals provided independent data which could be used as replicates within the 3‐hr time period. Determining discrete, independent time points within the three hours allowed for multiple replicates for each camera. Further, identifying the spatial independence between control and flowerpot treatments was necessary because of their relatively close proximity on the seawall (e.g., Favaro & Moore, 2015). Thus, both spatial and temporal serial autocorrelation was examined to determine how independent data were through time and from plot to plot on the wall (Appendix S1).

Following tests of serial autocorrelation (see Appendix S1), data were collected from 4 × 10‐min time points from two cameras, resulting in four replicates per camera in each location per sampling period. The 10‐min periods within the same camera were separated by 30 min of footage, and the periods between cameras at different points on the wall were separated by 10 min. Data were collected for the numbers of species and MaxN of each species, defined as the maximum number of individuals of a certain species present in one frame during the 10‐min time period (Cappo, Harvey, & Shortis, 2007). The results presented therefore measure the difference in the average maximum number of fish per treatment, rather than the average number of fish (hereafter termed abundance).

As outlined in Underwood (1993), asymmetrical analyses of variance were used to detect a difference between univariate measures of the fish assemblage at Blackwattle Bay and control locations. An effect of the treatment (i.e., presence of flowerpots) is shown as a difference in the temporal variability between treatment and control locations (if the effects vary through time) or through the main effect of treatment versus control locations (if the effects are temporally consistent) (Underwood, 1993). Fish were assigned to one of four functional groups on the basis of their trophic level (e.g., Guidetti, Fanelli, Fraschetti, Terlizzi, & Boero, 2002; Stuart‐Smith et al., 2013): (1) herbivores; (2) omnivores; (3) planktivores; and (4) predators. Allocation to groups was performed using online fish databases (Australian Museum and Fishes of Australia, Appendix S2). Benthic, habitat‐associated fish (from the families Blenniidae and Gobiidae) were excluded from analyses as a different camera deployment method is needed to sample these fish (see below).

The abundance of trophic groups along with species density (defined as the number of species per sample) was analyzed to test the null hypothesis that there would be no difference in any of the variables between seawalls with flowerpots and seawalls without flowerpots. Cochran's *C* test (Underwood, 1997) detected significant heterogeneity of variances, which could not be stabilized using a transformation. Analyses were therefore performed using untransformed data as analysis of variance is relatively robust to heterogeneous variances where the residual degrees of freedom are large (Sokal & Rohlf, 2012; Underwood, 1997).

To test the null hypothesis that species density and abundances of the trophic groups would not be different between sections of the wall with flowerpots and those without at Blackwattle Bay, standard symmetrical analyses of variance were used (three factors: habitat, fixed, two levels; time, random, 10 levels; site, random, two levels). Although there were no specific hypotheses about time, it was included as a random factor in the analyses to account for temporal variability in the fish assemblage. Variances were tested using Cochran's test, as before.

### 2.3 Benthic fish

As previously described, the flowerpots could be seen in the photographs taken with cameras deployed above the pots, whereas the surface of the seawall could not be viewed in the photographs taken by control cameras. The detection of fish closely associated with the substratum was therefore less likely in the control cameras in comparison with those deployed above flowerpots. In a second experiment, cameras facing downward to the substratum were used, which removed any confounding factor of not being able to detect benthic species. Therefore, any effects of flowerpots on benthic fish were only measured at the small scale, that is, within a seawall.

L‐shaped brackets were attached to the seawall at Blackwattle Bay, and cameras were bolted to the brackets in stainless steel housing during high tide (Figure 2b). Cameras were deployed in two treatments: (1) above flowerpots and (2) on adjacent control areas of seawall without flowerpots. One camera was deployed per treatment in the two sites. Due to the sedentary behavior of benthic fish, which meant that the same fish could be followed for the entire footage, the species and maximal abundance (using MaxN) of individuals was collected for 3 hr of footage. Time was therefore used as a replicate and was repeated to get a total of nine replicate sampling times between May and November 2015.

Species density was compared between treatments using a two factor analysis of variance (habitat, two levels: flowerpot and seawall, fixed and site, two levels: random and orthogonal). Abundance of benthic species was analyzed differently to pelagic fish as there were fewer species and the abundance of species was small, with many zeros in the dataset. Therefore, the difference between flowerpots and controls was further examined using χ^2^ calculations for the total number of individuals of each species separately (Chapman, 2012). Where the expected value was less than 5 in the χ^2^ calculations, an exact binomial test was used, which is robust to a small number of observations (Sokal & Rohlf, 2012).

## 3 RESULTS

Due to camera faults at some sampling times, there were seven sampling times for large‐scale effects, and 10 for small‐scale effects of flowerpots on pelagic fish. Data were also lost for one control and one flowerpot replicate in the benthic fish experiment. The ninth replicate for each treatment was averaged from the eight remaining replicates, and the degrees of freedom were adjusted accordingly in the analyses (Underwood, 1997).

A total of 24 pelagic fish species were sampled across all sampling times and locations (Appendix S2). These represented four trophic groups: predators (nine taxa); planktivores (eight taxa); omnivores (four taxa); and herbivores (three taxa). Predatory fish were those that feed on benthic invertebrates; no piscivores were observed. Seven benthic fish species were recorded from the families Blenniidae (three taxa), Gobiidae (three taxa) and Tripterygiidae (one taxon). Juveniles of three taxa were also identified; *Acanthopagrus australis*,* Centropogon australis*, and one unidentified species of leatherjacket (Monocanthidae).

### 3.1 Large‐scale effects on pelagic fish

**Figure 3:**
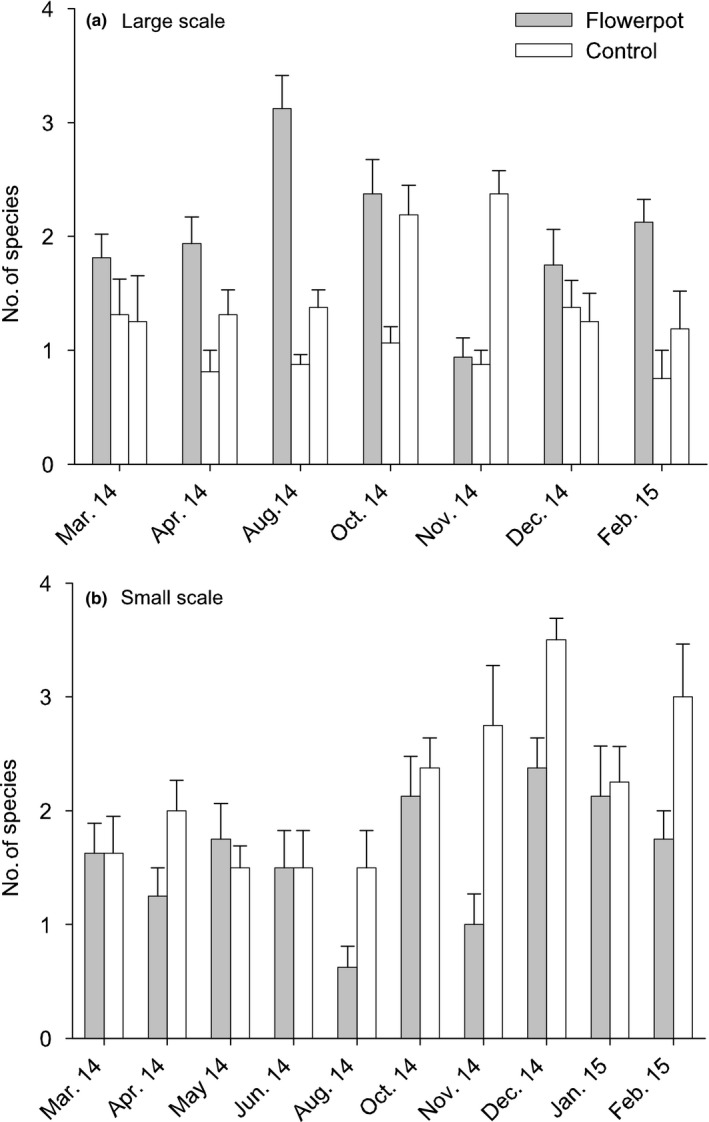
Mean (±*SE*) species density of pelagic fish at two spatial scales: (a) large, at the location where flowerpots were installed (Blackwattle Bay, gray bars) compared to control locations without flowerpots (Balmain, middle bar; North Sydney, right‐hand bar; white bars) at seven sampling times (*n* = 8) and (b) small, areas of the seawall with (gray bars) and without (white bars) flowerpots at Blackwattle Bay at ten sampling times (*n* = 4). See Appendices S5 and S6 for graphs of medians and ranges

**Figure 4:**
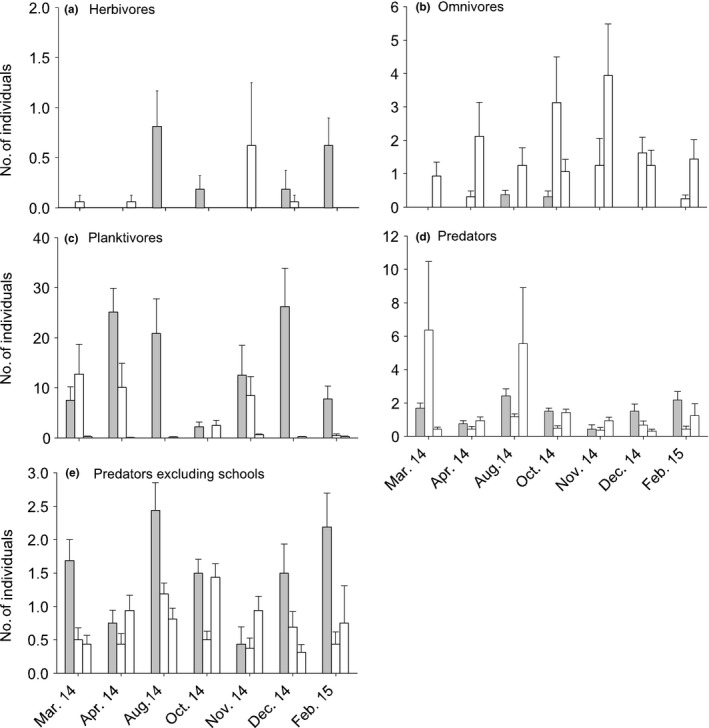
Mean (±*SE*) abundance of pelagic fish at Blackwattle Bay (with flowerpots, gray bars) and two control locations (Balmain, middle bar; North Sydney, right‐hand bar; white bars) over seven sampling times (*n* = 8). Note the different scales on the *y*‐axis. See Appendix S5 for graphs of medians and ranges

There were no significant main effects of adding flowerpots to seawalls for any of the variables of the fish assemblage measured (Table 1, Figures 3a and 4a–e). Species density varied significantly through time from site to site in the control locations, but not at Blackwattle Bay (significant *T* × *S* (C) interaction, Table 1), indicating that the flowerpots may have reduced some measure of variability between sites. Herbivores, in contrast, showed interaction at the larger scale, with greater abundances at Blackwattle Bay in August, October and February, but not at other times (significant *T* × BW vs. C, Table 1; Figure 4a).

**Table 1:** Summary of significant *F*‐ratios from large‐ and small‐scale analyses. Details of these analyses are given in Appendices S3 and S4

Source of variation	Species density	Herbivore	Omnivore	Planktivore	Predator	Predator exc. schools
*(a) Large scale*						
Time, *T*						
Locations, *L*						
BW vs. C	ns		ns		ns	
Between C	ns		ns		ns	
Site(*L*), S(*L*)						
S(BW)	ns		*		***	
S(C)	***		ns		ns	
*T* × *L*						
*T* × BW vs. C	ns	***	ns	**	ns	*
*T* × Between C	ns	ns	ns	ns	ns	ns
*T* × S(*L*)						
*T* × S(BW)	ns	ns	***	*	*	***
*T* × S(C)	*	ns	***	ns	ns	ns
(b) Small scale						
Time, *T*	ns	ns		ns		ns
Site, *S*	ns	ns		ns		ns
Treatment, Tr	No test	ns		No test		No test
*T* × *S*	***	***		**		***
Tr × *T*	ns	ns		ns		ns
Tr × *S*	ns	ns		ns		ns
Tr × *T* × *S*	ns	ns	***	ns	***	ns

*p < .05, **p < .01, ***p < .001.

There was significant temporal variability among sites at Blackwattle Bay and in control locations for the abundance of omnivores, although no effect of adding flowerpots to the seawall was seen (Table 1, Figure 4b). On the contrary, planktivores showed interaction at the larger scale, with greater abundances at Blackwattle Bay in general, although this effect was variable over time (significant *T* × BW vs. C interaction, Table 1; Figure 4c).

There were no significant differences between experimental and control locations for predators (Table 1). The majority of predators were individuals or in small groups, such as *Acanthopagrus australis* and *Tetractenos hamiltoni*. In a few replicates, large schools of Atherinidae were seen, causing greater variability in the control sites (Figure 4d). Removing *Atherinomorus vaigiensis* and *Atherinosoma microstoma* from the analysis of predators showed a similar pattern to planktivorous species where the abundance of predators tended to be greater at the wall with flowerpots, although this was not consistent at all sampling times (significant *T* × BW vs. C interaction, Table 1; Figure 4e).

### 3.2 Small‐scale effects on pelagic fish

**Figure 5:**
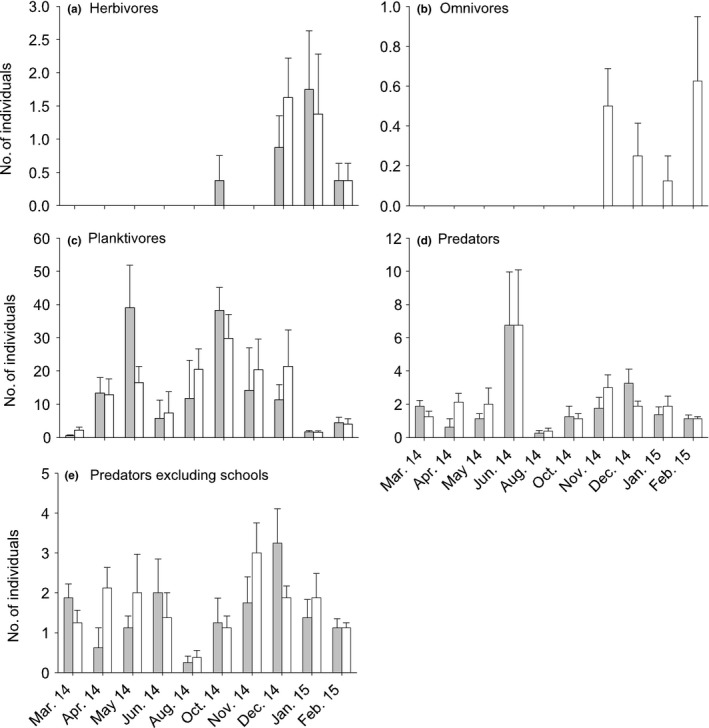
Mean (±*SE*) abundance of pelagic fish at Blackwattle Bay in areas of the seawall with (gray bars) and without (white bars) flowerpots at 10 sampling times (*n* = 4). Note the different scales on the *y*‐axis. See Appendix S6 for graphs of medians and ranges

Mean species density appeared to be similar between flowerpots and seawall at most sampling times (Figure 3b). A test was not, however, possible, for the main effect of habitat on species density because lower order interactive effects could not be pooled (Underwood, 1997), although they were not significant (Table 1). Similarly, there was no effect of treatment on herbivores (Table 1, Figure 5a).

The abundance of omnivores was greater in areas of the seawall without, compared to with, flowerpots at both sites in November, and in sites 2 and 1 in December and February, respectively (significant Tr × *T* × *S* interaction, Table 1; Figure 5b). This was due to luderick, *Girella tricuspidata*, and fanbelly leatherjacket, *Monacanthus chinensis*, being found only at areas without pots; no differences were found at other sampling times. Again, no test was possible for the effect of treatment on planktivores, although there were no clear patterns of difference between treatments over time (Table 1, Figure 5c).

There was a significantly greater abundance of predatory fish at areas of the seawall without flowerpots in site 1; however, the opposite was found at site 2 in June, but not at other times (significant Tr × *T* × *S* interaction, Table 1; Figure 5d). This result was driven by large schools of *Atherinomorus vaigiensis*. Therefore, as before, analyses were performed excluding this species and there was no significant difference between treatments for the abundance of predators excluding these schools (Table 1, Figure 5e).

### 3.3 Small‐scale effects on benthic fish

When benthic species were measured using downwards facing cameras, there was no significant difference in species density of benthic fish (*F*_1,1_ = 0.51, *p* > .05) between flowerpot and control treatments. In contrast, the total abundance of the rotund blenny, *Omobranchus rotundiceps*, was significantly greater at sites with flowerpots than in control areas of the seawall (Table 2). Although not significant, the total number of the oyster blenny, *Omobranchus anolius*, appeared to be greater in control than flowerpot treatments (Table 2). No other significant differences were found for any other species (Table 2).

**Table 2:** χ^2^ tests for the total abundance of benthic fish taxa found at flowerpots and control seawall treatments over ten sampling times (*df* = 1). Where the expected value was less than 5 in the χ^2^ calculations, an exact binomial test was used

	Flowerpot	Seawall	χ^2^	Binomial
*Omobranchus anolius*, Oyster blenny	15	22	ns	
*Omobranchus rotundiceps*, Rotund blenny	12	1		a
*Parablennius intermedius*, Horned blenny	2	2		ns
*Redigobius macrostoma*, Largemouth goby	7	8		ns
*Bathygobius cocosensis*, Cocos frillgoby	2	2		ns
*Cryptocentroides gobioides*, Oyster goby	0	1		ns
*Enneapterygius atrogulare*, Ringscale triplefin	2	0		ns
*Acanthopagrus australis* juvenile	11	6	ns	
*Centropogon australis* juvenile	1	1		ns
Leatherjacket juvenile	1	1		ns

a Significant effect.

Juvenile *Acanthopagrus australis* close to the seawall were included in the χ^2^ tests because, although not a cryptic fish, they are likely to have an association with the substratum for shelter and were found more often in flowerpots in the first experiment. A pattern for a greater number of juveniles was still found at the flowerpots in comparison with control areas of the seawall, although this was not significant (Table 2). Only two other juvenile taxa were seen, both as singletons in the control and flowerpot treatment: *Centropogon australis*; and an unidentified leatherjacket (Table 2).

## 4 DISCUSSION

There was no consistent effect of flowerpots on the fish assemblage over the year of this study (Table 3). At the larger scale of a few km (Figure 1), planktivores and predators tended to be more abundant at the seawall with flowerpots, although this was temporally variable. Equally, herbivores were also more abundant at Blackwattle Bay at three of the seven sampling times, although during the other times there were no significant differences in abundance. We observed few differences in diversity or abundance of pelagic fish at the smaller spatial scale. Omnivores were more abundant in control areas at certain sites at three of the 10 sampling times only. Similarly, the number of benthic species was not significantly different in areas of the seawall with or without flowerpots, although there were some species‐specific responses.

**Table 3:** Summary of results. Significant effects of treatment are in bold. S = significant, NS = nonsignificant, *T* = interaction with time, *T*,Si =interaction with time and site

	Pelagic						Benthic										
	Pelagic species density	Herbivore	Omnivore	Planktivore	Predator	Predator exc. schools	Benthic species density	*Omobranchus anolius*	*Omobranchus rotundiceps*	*Parablennius intermedius*	*Redigobius macrostoma*	*Bathygobius cocosensis*	*Cryptocentroides gobioides*	*Enneapterygius atrogulare*	*Acanthopagrus australis*	*Centropogon australis* juv.	Leatherjacket juvenile
Large scale	NS	**T**	NS	**T**	NS	**T**											
Small scale	NS	NS	**T,Si**	NS	**T,Si**	NS	NS	NS	**S**	NS	NS	NS	NS	NS	NS	NS	NS

A frequent limitation of eco‐engineering research is the lack of replicability of experiments at multiple locations (e.g., Toft et al., 2013; but see Browne & Chapman, 2014), although experiments have been replicated at sites within a single location (e.g., Chapman & Blockley, 2009). This is often due to the permission required to alter built infrastructure at multiple locations. It does complicate and reduce rigorous interpretation of such large‐scale managerial experiments (Chapman et al., 2017). The use of asymmetrical designs does, however, allow complex designs in which there is only one experimental location and multiple control locations to be analyzed (Underwood, 1993). They therefore allowed effects of eco‐engineered habitats among seawalls to be tested in this study.

Although we are limited in the generalizations we can make beyond the location used, we have provided correlative evidence that the fish assemblage at the location with flowerpots installed was different from the average of two control locations. This was due to a significantly different temporal variation in herbivores, planktivores, and predators at seawalls with flowerpots (Table 3). Repeating this experiment in multiple locations would provide strength to this conclusion (Glasby, 1997). In addition, collection of data before the pots were deployed, in addition to afterward, would have provided the necessary evidence that these differences developed coincident with the installation of the pots. Unfortunately, there was not enough time between permission being granted to install the flowerpots and deployment to collect such data, which is often the case in studies that require collaborations with managers of urban infrastructure. Nevertheless, this study is building on the limited knowledge we have on the effect of habitat enhancements on fish communities associated with artificial structures other than artificial reefs in the marine environment (Munsch, Cordell, & Toft, 2017; Sella & Perkol‐Finkel, 2015; Toft et al., 2013).

The change in habitat complexity and/or biodiversity caused by ecological engineering of seawalls for benthic species may thus have knock‐on effects for fish assemblages. Equally, an induced change in the fish assemblage could have consequences for the organisms living on artificial structures. The addition of complex surfaces and novel habitats (e.g., the flowerpots) to marine infrastructure has resulted in an increase in the number and/or abundance of benthic species living on that structure (e.g., Browne & Chapman, 2014; Chapman & Underwood, 2011; Firth et al., 2014). Similarly, at a smaller spatial scale of a few hundred meters, previous experiments showed increased diversity and/or abundances of fish in response to eco‐engineered habitats in comparison with the adjacent unmodified structure (Sella & Perkol‐Finkel, 2015; Toft et al., 2013). Those increases were not replicated in this study. This could be due to the different scale at which the enhancements were made. For instance, Sella and Perkol‐Finkel (2015) deployed eco‐engineered breakwater units that were 1 m^3^ and up to 2.5 tonnes, thus a lot bigger than the flowerpots deployed here. Similarly, in the United States, seawalls designed specifically for fish (in particular salmon) incorporated habitats over hundreds of meters along the seawall (Toft et al., 2013).

A recent review described an example of another large‐scale eco‐engineering project in the United States, currently in the process of being built (Munsch et al., 2017). The need for a seawall upgrade presented the opportunity to enhance the habitat for juvenile salmon, and the invertebrates they feed on. Enhancements included complex habitat enhancement panels to increase epibenthic prey, marine mattresses to create nearshore shallow water habitat and light‐penetrating panels to facilitate greater use of areas under piers by juveniles (Munsch et al., 2017). This review highlighted that few eco‐engineering designs had been evaluated in terms of fish habitat, including rock pools on seawalls. Here, we present the first data on the effect of rock pools on seawalls, but at this small scale, there may be few effects on fish assemblages (although they have an effect on benthic communities, which is why they were added). Thus, enhancements for fish may be more successful at the seawall scale being implemented in other locations (e.g., the United States). It is difficult to predict whether there would be a different response of fish to a seawall supporting 100s of meters of flowerpots. Managerial decisions need to be made on existing evidence due to the difficulties of doing large, well‐replicated experiments in urbanized harbors (Chapman et al., 2017), precisely where such experiments are most needed (Chapman & Underwood, 2011). Adaptive management allows such decisions to be modified as new data come to light (Thom, 2000; Walters & Holling, 1990).

This study extended our current understanding by testing the effects of eco‐engineering for benthic diversity on fish assemblages at two spatial scales; among walls with or without flowerpots km apart and between areas of the same seawall with or without these added habitats. Abundances of herbivores, planktivores, and predators were greater in the waters around the seawall with flowerpots, compared to controls, although this was temporally variable, but this did not occur at a smaller scale within a site. This indicates that the fish responded to walls with flowerpots, but at that wall, did not respond to the flowerpots at all. This complex result, a different result at the small and at the large scale, supports research that has shown the importance of assessing habitat quality for fish assemblages at multiple spatial scales (Harborne, Mumby, Kennedy, & Ferrari, 2011; Johnson, Jenkins, Hiddink, & Hinz, 2013). For instance, Harborne et al. (2011) found that coral‐reef‐associated fish were more abundant on refuge‐rich and taller corals at a colony scale, but abundance was also positively correlated at a comparatively larger scale with the number of colonies within an area. Alternatively, as previously discussed, these results may or may not be coincident with the installation of the flowerpots.

For predators, eco‐engineered habitats may provide a greater abundance of prey. For example, in Seattle, prey availability and juvenile salmon feeding frequency were greater at a created beach compared to artificial riprap habitat (Munsch, Cordell, & Toft, 2015a; Toft et al., 2013). Similarly, these flowerpots were particularly successful in increasing the abundance of algae (Morris, unpublished data), which could provide food for herbivorous fish. Where eco‐engineering increases the abundance of predatory or herbivorous fish, this could have an effect on the success of these habitats for benthic species. Controlled, manipulative experiments are needed to directly test the effects of fish predation or herbivory on developing benthic assemblages (Anderson & Connell, 1999; Hixon & Brostoff, 1996). Conversely in areas where there are large numbers of predatory fish, certain eco‐engineered features may provide a refuge for intertidal species (Strain, Morris et al., 2017). Planktivores have been observed in greater numbers around artificial structures that span the entire water column (Rilov & Benayahu, 1998). Whilst the association between flowerpots and planktivores is less clear, previous research has shown a positive correlation between the complexity of oil jetty pillars and the abundance of plankton feeders, possibly due to increased shelter from predation (Rilov & Benayahu, 1998).

Counter to predictions, there was not an overall difference in the number of benthic fish species in areas of the seawall with flowerpots than without. This was predicted as cryptic fish, such as blennioid assemblages, can be characterized at fine scales by topographic features (Syms, 1995). The results showed, however, that similar cryptic fish were found in areas of the seawall with or without flowerpots, although the abundance of certain species differed in the two types of area. There were a significantly greater number of rotund blennies (*Omobranchus rotundiceps*) in areas with flowerpots. Although little information was available regarding this species’ habitat preferences, the rotund blenny is frequently seen in rock pools on natural shores in the area (pers. obs.), and therefore, it may not be surprising that it responded to artificial rock pools. In contrast, the oyster blenny (*Omobranchus anolius*) was more abundant on the seawall without pots but with extensive cover of oysters than at the flowerpots, although this difference was not significant. No other differences were seen in the number or identity of benthic species between the two types of habitat. One reason for this may be because the oyster bed on the seawall provided the microhabitat needed by cryptic species. This result highlights that where we add complexity to seawalls, a portion of the fish assemblage that interacts directly with that complexity (e.g., it provides a habitat similar to fishes’ natural preferences) may respond. Notably, however, this means that habitat enhancements can provide habitat for some species, at the expense of habitat for others. For instance, in this case, flowerpots created habitat to which rotund blennies may respond, whilst precluding oyster habitat to which oyster blennies respond. This raises the important question about how we decide what taxa to enhance, which should be set out in specific management objectives at the beginning of the project (see Mayer‐Pinto et al., 2017).

Despite urbanized systems being heavily degraded, fish still utilize them, and their habitat value is often unclear. Management initiatives to enhance benthic intertidal species living on seawalls could have knock‐on effects on fish assemblages, and effects may be greater if eco‐engineering is performed on a comparatively larger scale. Thus, it is important to evaluate ecosystem‐wide effects to fully understand the consequences of eco‐engineering. Notably, there was little effect of flowerpots on the fish assemblage at the size and spatial scale that they were deployed here for benthic species. Successes of eco‐engineering are more prevalent in the literature, but arguably more can be learnt from what does not work (Firth, Browne et al., 2016). Eco‐engineering is growing, and many decisions and a lot of money will be spent in the future based on the published literature (Chapman et al., 2017). Thus, for projects targeting fish enhancement, we have communicated important information on what may, and may not, be successful. Whether enhancement of coastal structures could be used to support viable populations of fish is still a question that remains. Further studies to provide a link between the different abiotic and biotic factors affecting fish species associated with ecologically enhanced infrastructure could provide insight into optimizing habitat design if the target group included (or excluded) fish. The spatial scale to which species respond, and understanding the response of assemblages at multiple spatial scales is an essential consideration when manipulating these artificial habitats.

## CONFLICT OF INTEREST

None declared.

## AUTHOR'S CONTRIBUTIONS

RLM, RAC, and MGC conceived the ideas and designed the methodology; RLM collected and analyzed the data; RLM, RAC, MGC, and LBF interpreted the data and wrote the manuscript. All authors contributed critically to the drafts and gave final approval for publication.

## DATA ACCESSIBILITY

If the manuscript is accepted for publication, the data will be made available at The University of Sydney's data repository (https://ses.library.usyd.edu.au/).
