# Supplementary material for: Increasing habitat complexity on seawalls: Investigating large‐ and small‐scale effects on fish assemblages
Source: Ecol Evol. 2017 Oct 14;7(22):9567–79. doi: 10.1002/ece3.3475 (PMC5696408; doi:10.1002/ece3.3475)
Supplement: Supplementary file 1 [file ECE3-7-9567-s001.docx]

**Appendix S1:** Methods and results for autocorrelation

S1.1 Method

Serial autocorrelation was done on data collected from cameras at Blackwattle Bay for four randomly selected months. The total footage was split into nine x 10 min intervals, each separated by 10 min. Data on the species and abundance (using Max N, Cappo *et al*. 2007) of fish were collected from each 10 min period. Ten minutes was chosen to get enough replicate time periods within the 3 hours of footage, and is comparable to previous studies deploying unbaited cameras (Watson *et al*. 2005). The variables used for the serial autocorrelations were abundance of all taxa and diversity using Shannon-Weiner diversity index. A measure of diversity was used to take into account the probability that two individuals from different time periods or treatments would belong to different species (Favaro & Moore 2015). Temporal serial autocorrelations were done separately for control and flowerpot cameras.

Using the same method as Underwood and Chapman (1996), serial autocorrelations were calculated for all time periods between pairs of 10 min intervals from footage taken by the same camera, hereafter referred to as separations. The separation between time intervals varied from 1 (adjacent time periods) to 4, which is half the total number of time intervals (Underwood & Chapman 1996). To determine correlation between adjacent cameras, again serial autocorrelations were calculated for each separation. The separation however, varied from 0 (the same time period) to 4. The significance for each separation of time was determined from 1000 randomisations of the data, with the probability level set at 5% (Underwood & Chapman 1996). To determine whether tidal state influenced the variability of the four replicate time periods used within the 3 hour deployment, a two-way analysis of variance on the data from Blackwattle Bay with tidal period (fixed, 4 levels) and treatment (fixed, 2 levels) was done. If the tidal state had an influence, we expected to see patterns in the abundance of fish from the 1^st^, 2^nd^, 3^rd^ and 4^th^ 10 minute period.

S1.2 Results

Temporal autocorrelation among time intervals gave few significant correlations for both variables in the control and flowerpot treatments (Figure S1.1a-d and Table S1.1). Spatially, serial autocorrelation showed the highest number of significant correlations between adjacent control and flowerpot treatments at a time interval of zero (Table S1.1 and Figure S1.1e, f). As there was no pattern of correlation between the time intervals tested within cameras, these were therefore considered independent. Similarly, time intervals between cameras at a separation of 1 or more showed no general trend of correlation and were also considered independent. Because temporal and spatial independence was identified, this enabled replication within time points Further, there was no significant effect of tidal period (ANOVA, F_3,155_ = 1.18, P>0.05, n=20), thus suggesting that using multiple replicates within a camera deployment did not have an effect on the variability of the data.

S1.3 References

Cappo, M., Harvey, E., Shortis M. (2007) Counting and measuring fish with baited video techniques - an overview. *Proceedings of the Australian Society for Fish Biology Workshop, Hobart, August 2007* (eds D. Furlani & J.P. Beumer), pp. 101-114. Australia.

Favaro, C. & Moore, J.W. (2015) Fish assemblages and barriers in an urban stream network. *Freshwater Science,* **34,** 991-1005.

Underwood, A.J. & Chapman, M.G. (1996) Scales of spatial patterns of distribution of intertidal invertebrates. *Oecologia,* **107,** 212-224.

Watson, D.L., Harvey, E.S., Anderson, M.J. & Kendrick, G.A. (2005) A comparison of temperate reef fish assemblages recorded by three underwater stereo-video techniques. *Marine Biology* **148**, 415-425.


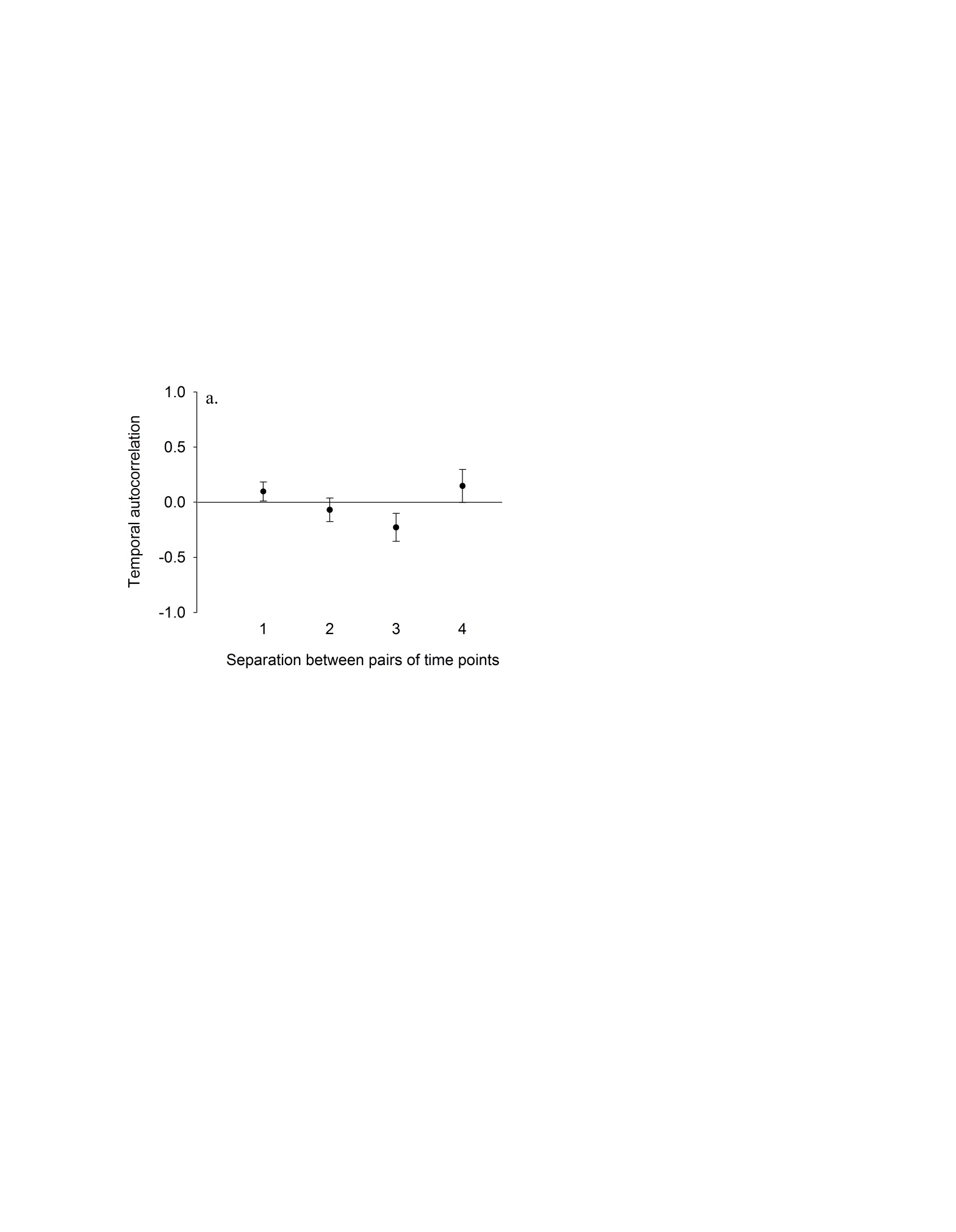

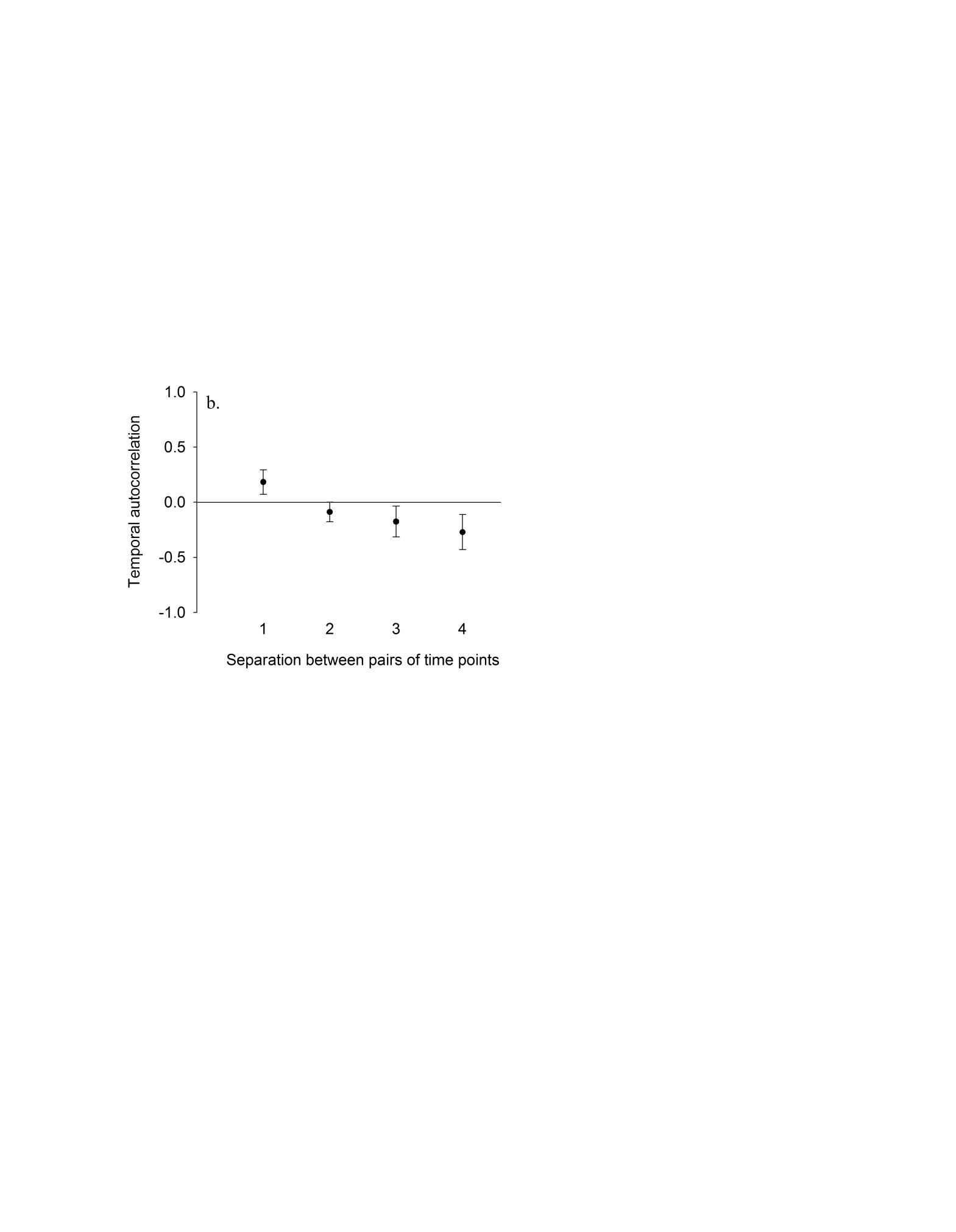

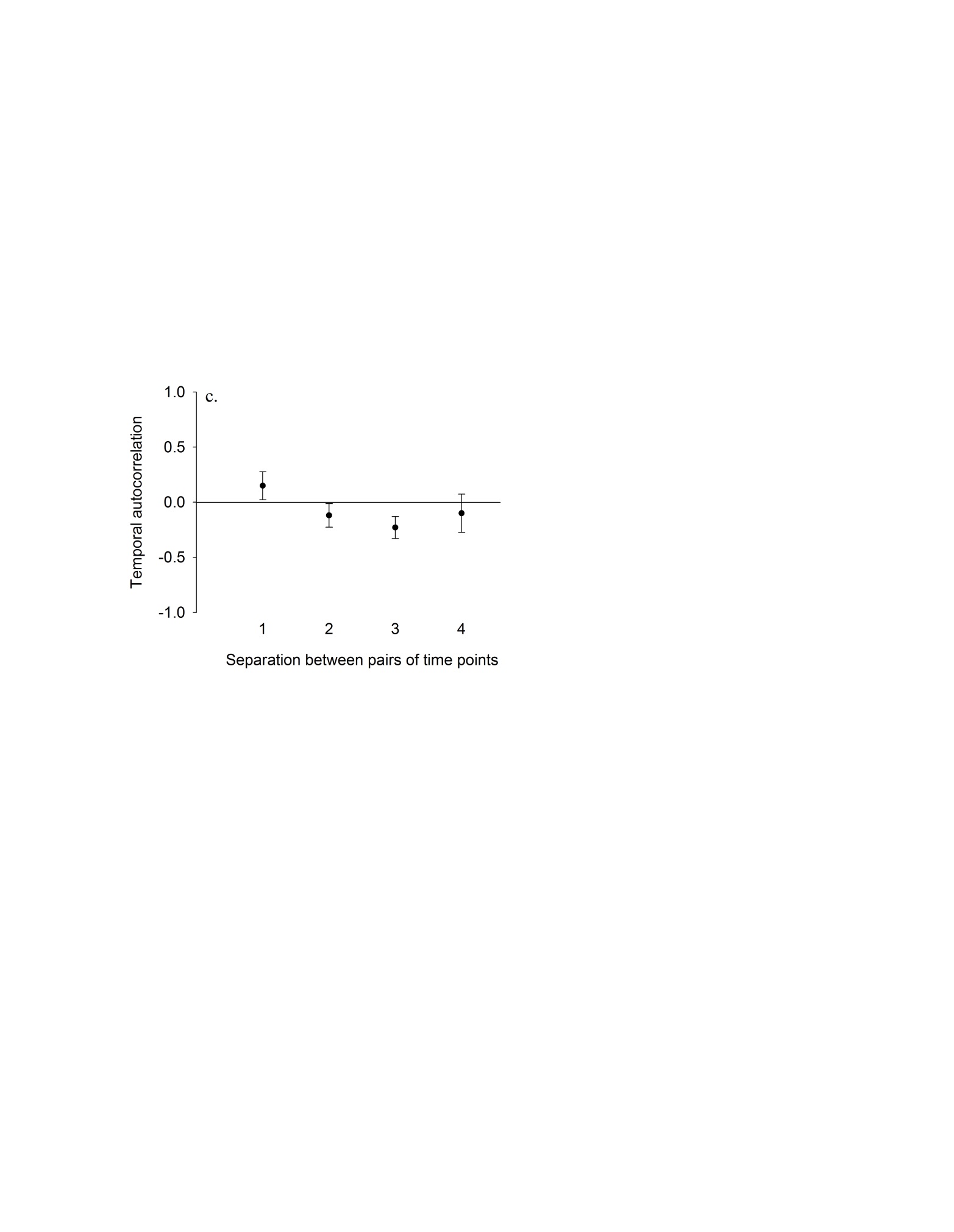

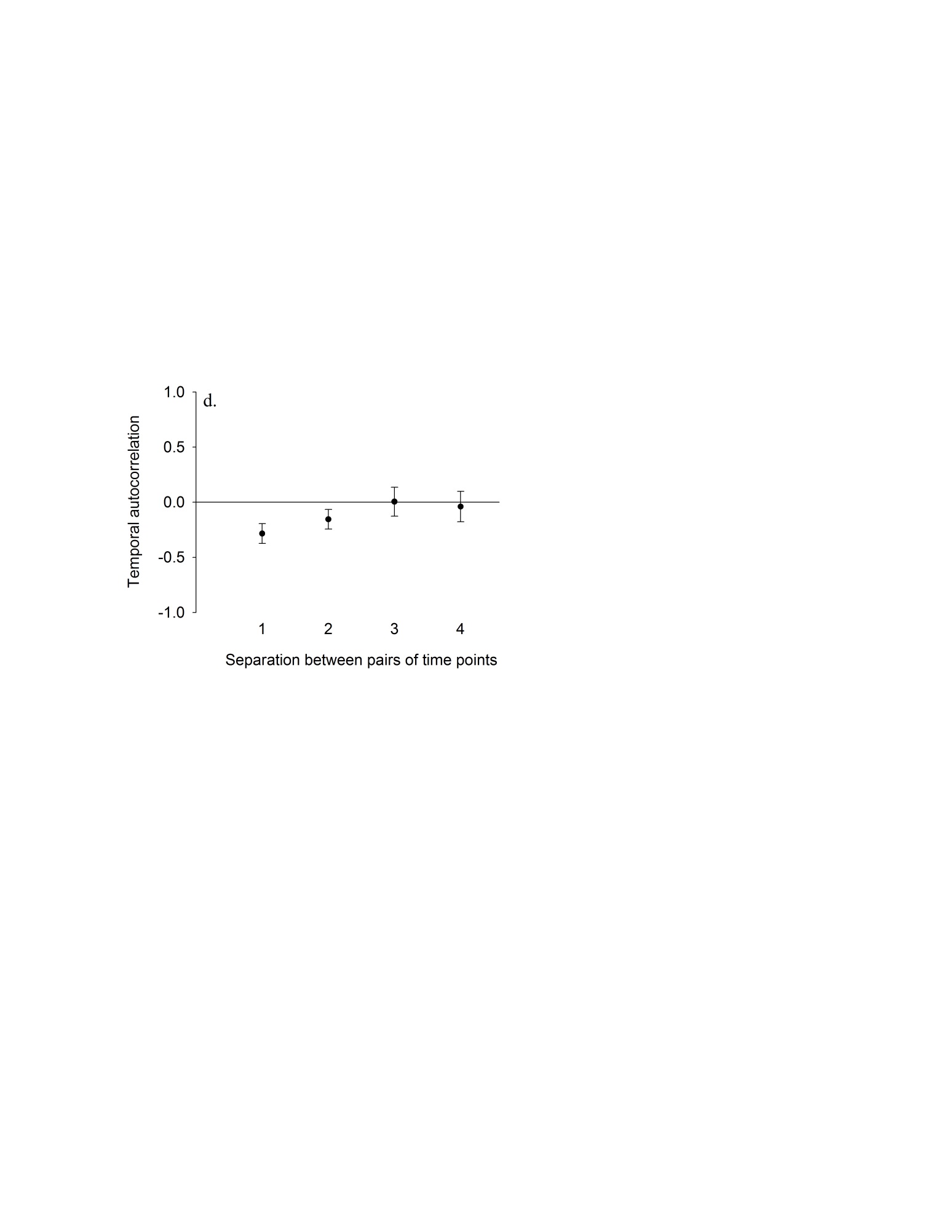

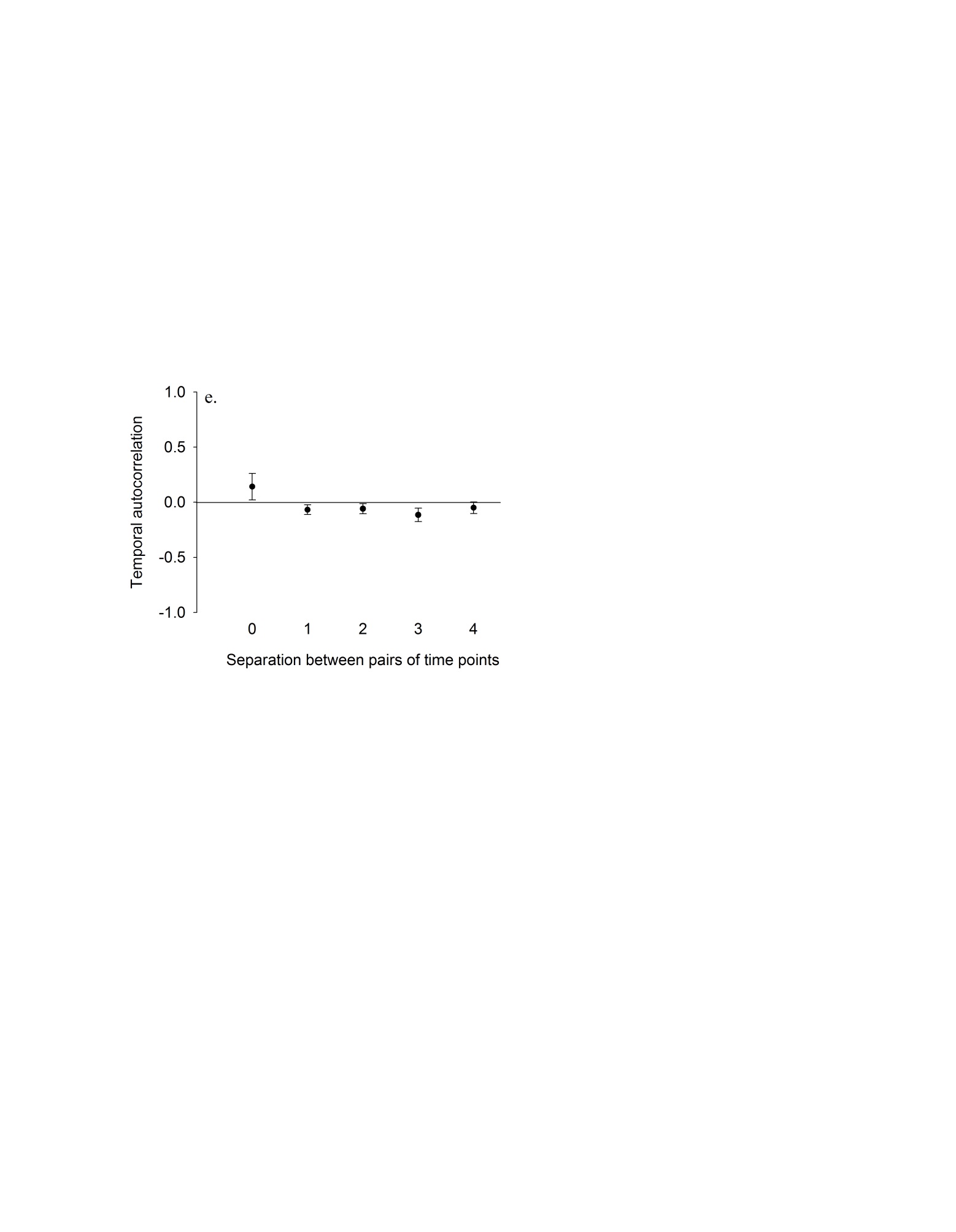

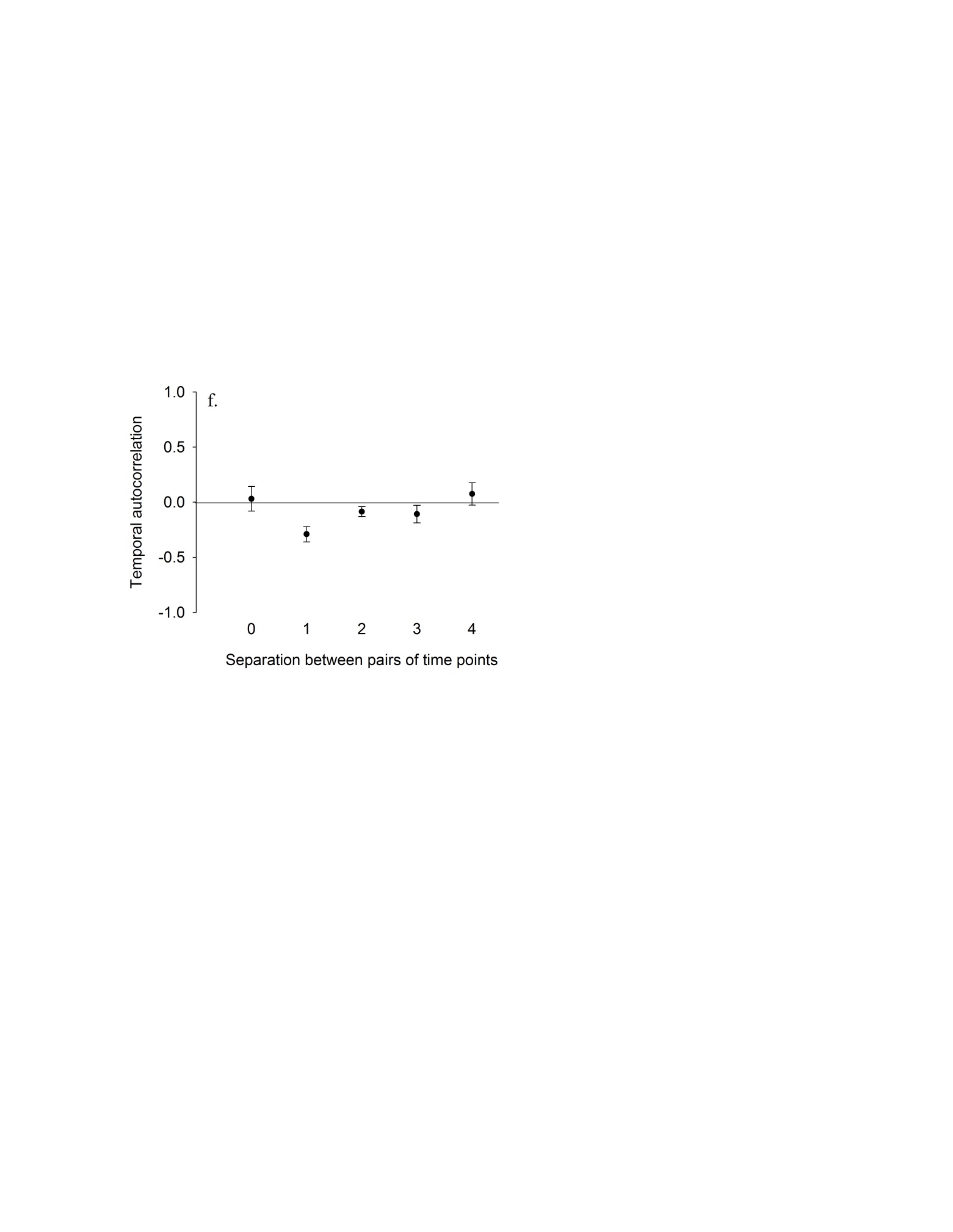


**Fig. S1.1**: Serial autocorrelation (± S.E.) between pairs of time intervals separated by 1 (adjacent intervals) to 4 (half the number of intervals) for a. total abundance of fish in control treatment, b. Shannon’s Diversity of control treatment, c. total abundance of fish in flowerpot treatment and d. Shannon’s Diversity of flowerpot treatment. Serial autocorrelation between pairs of time intervals separated by 0 (same time interval) to 4 (half the number of intervals) for e. total abundance of fish in adjacent treatments and f. Shannon’s Diversity of adjacent treatments. A value of 0 on the y axis indicates no correlation.

**Table S1.1**: Number of cameras with a significant correlation determined from serial autocorrelation between time points separated by different lag times.

| **Treatment** | **Measurement** | **Direction of correlation** | **Separation between time intervals** | | | | |
| --- | --- | --- | --- | --- | --- | --- | --- |
|  |  |  | **0** | **1** | **2** | **3** | **4** |
| Control | Total abundance | positive |  |  |  |  |  |
| N=13 |  | negative |  |  |  | 1 |  |
|  | Shannon’s Diversity | positive |  |  |  |  |  |
|  |  | negative |  |  |  |  | 2 |
| Flowerpots | Total abundance | positive |  |  |  |  |  |
| N=10 |  | negative |  |  |  |  | 1 |
|  | Shannon’s Diversity | positive |  |  |  |  |  |
|  |  | negative |  |  |  |  |  |
| Space | Total abundance | positive | 3 |  |  |  |  |
| N=16 |  | negative |  |  |  |  |  |
|  | Shannon’s Diversity | positive | 2 |  |  |  | 1 |
|  |  | negative | 1 | 5 |  | 2 |  |

**Appendix S2**: List of fish taxa and the assignment of functional groups based on trophic level.

| Taxa | Trophic level |
| --- | --- |
| *Abudefduf sexfasciatus* Scissortail sergeant | Planktivore |
| *Acanthopagrus australis* Yellowfin bream | Predator |
| *Acanthopagrus australis* Yellowfin bream juvenile | Predator |
| *Achoerodus viridis* Eastern Blue Groper | Predator |
| *Ambassis jacksoniensis* Port Jackson Glassfish | Planktivore |
| *Ambassis marianus* Estuary Glassfish | Planktivore |
| *Atherinomorus vaigiensis* Common Hardyhead | Predator |
| *Atherinosoma microstoma* Small mouth Hardyhead | Predator |
| Garfish sp. | Herbivore |
| *Brachaluteres jacksonianus* Southern pygmy leatherjacket | Predator |
| *Dicotylichthys punctulatus* Threebar porcupinefish | Predator |
| *Gerres subfasciatus* Common silverbiddy | Predator |
| *Girella elevata* Rock blackfish | Omnivore |
| *Girella tricuspidata* Luderick | Omnivore |
| *Gobiopterus semivestitus* Glassgoby | Planktivore |
| *Hyperlophus vittatus* Sandy sprat | Planktivore |
| *Microcanthus strigatus* Stripey | Omnivore |
| *Monacanthus chinensis* Fanbelly leatherjacket | Omnivore |
| *Mugil cephalus* Sea mullet | Herbivore |
| *Odax cyanomelas* Herring cale | Herbivore |
| *Pandaka lidwilli* Lidwills Dwarfgoby | Predator |
| *Parioglossus marginalis* Blackmargin dartfish | Planktivore |
| *Scorpis lineolata* Silver sweep | Planktivore |
| *Scorpis lineolata* Silver sweep Juvenile | Planktivore |
| *Tetractenos hamiltoni* Common toadfish | Predator |
| *Trachurus novaezelandiae* Yellowtail Scad | Planktivore |

**Appendix S3**: Asymmetrical analyses of variance testing the difference between seven measures of the fish assemblage where flowerpots were installed (Blackwattle Bay) and two control locations (Balmain and Pyrmont) at seven times of sampling after pot deployment (n = 8). ‘Sites’ are nested in ‘Locations’, therefore are not a direct test of a difference between Blackwattle Bay and the control locations. A test of T × S(C) and S(C) was done to determine if these terms could be pooled. A 2-tailed F-test at the scale of sites tested whether the variation between sites at Blackwattle Bay was different to that among sites at control locations. A test for the main effect of BW vs C directly tested the hypothesis of a greater number of species and/or abundance of fish at Blackwattle Bay compared to control locations.

| **Species density**, Cochran’s test, C = 0.11, P < 0.01 | | | | | | | |
| --- | --- | --- | --- | --- | --- | --- | --- |
| Source of variation | df | | MS | | F | P | F vs. |
| Time, T | 6 | | 4.39 | |  | |  |
| Locations, L | 2 | | 28.11 | |  |  |  |
| BW vs C | 1 | | 39.05 | | 2.26 | 0.27 | S(C) |
| Between C | 1 | | 17.16 | | 0.99 | 0.42 | S(C) |
| Site(L), S(L) | 3 | | 11.87 | |  |  |  |
| S(BW) | 1 | | 1.08 | | 15.98 | 0.06 | S(C)/S(BW) |
| S(C) | 2 | | 17.26 | | 10.43 | 0.00 | T × S(C) |
| T × L | 12 | | 3.47 | |  |  |  |
| T × BW vs C | 6 | | 4.17 | | 1.50 | 0.32 | T × Bet C |
| T × Between C | 6 | | 2.78 | | 1.68 | 0.21 | T × S(C) |
| T × S(L) | 18 | | 2.21 | |  |  |  |
| T × S(BW) | 6 | | 3.33 | | 2.01 | 0.14 | T × S(C) |
| T × S(C) | 12 | | 1.65 | | 2.13 | 0.02 | Res |
| Residual | 294 | | 0.78 | |  |  |  |
|  | | | | | | | |
| **Herbivores**, Cochran’s test, C = 0.25, P < 0.01 | | | |  | |  |  |
| Source of variation | df | | MS | F | | P | F vs. |
| Time, T | 6 | | 1.17 |  | |  |  |
| Locations, L | 2 | | 1.78 |  | |  |  |
| BW vs C | 1 | | 3.01 |  | |  |  |
| Between C | 1 | | 0.54 |  | |  |  |
| Site(L), S(L) | 3 | | 0.34 |  | |  |  |
| S(BW) | 1 | | 0.44 |  | |  |  |
| S(C) | 2 | | 0.29 |  | |  |  |
| T × L | 12 | | 1.54 |  | |  |  |
| T × BW vs C | 6 | | 2.63 | 5.53 | | 0.00 | Res |
| T × Between C | 6 | | 0.45 | 0.94 | | 0.47 | Res |
| T × S(L) | 18 | | 0.39 |  | |  |  |
| T × S(BW) | 6 | | 0.19 | 2.60 | | 0.07 | T × S(C)/T × S(BW) |
| T × S(C) | 12 | | 0.49 | 1.03 | | 0.42 | Res |
| Residual | 294 | | 0.48 |  | |  |  |
| Appendix S3 cont. | | | |  | |  |  |
| **Omnivores**, Cochran’s test, C = 0.28, P < 0.01 | | | |  | |  |  |
| Source of variation | df | | MS | F | | P | F vs. |
| Time, T | 6 | | 13.82 |  | |  |  |
| Locations, L | 2 | | 73.16 |  | |  |  |
| BW vs C | 1 | | 112.54 | 4.35 | | 0.06 | T × S(C) |
| Between C | 1 | | 33.79 | 1.31 | | 0.28 | T × S(C) |
| Site(L), S(L) | 3 | | 7.60 |  | |  |  |
| S(BW) | 1 | | 0.08 | 141.31 | | 0.01 | T × S(C)/T × S(BW) |
| S(C) | 2 | | 11.36 | 0.44 | | 0.65 | T × S(C) |
| T × L | 12 | | 12.73 |  | |  |  |
| T × BW vs C | 6 | | 6.09 | 0.24 | | 0.96 | T × S(C) |
| T × Between C | 6 | | 19.37 | 0.75 | | 0.62 | T × S(C) |
| T × S(L) | 18 | | 17.35 |  | |  |  |
| T × S(BW) | 6 | | 0.29 | 89.66 | | 0.00 | T × S(C)/ T × S(BW) |
| T × S(C) | 12 | | 25.88 | 5.28 | | 0.00 | Res |
| Residual | 294 | | 4.91 |  | |  |  |
|  | | | |  | |  |  |
| **Planktivores**, Cochran’s test, C = 0.21, P < 0.01 | | | |  | |  |  |
| Source of variation | | df | MS | F | | P | F vs. |
| Time, T | | 6 | 541.27 |  | |  |  |
| Locations, L | | 2 | 5850.12 |  | |  |  |
| BW vs C | | 1 | 10824.12 |  | |  |  |
| Between C | | 1 | 876.11 |  | |  |  |
| Site(L), S(L) | | 3 | 1379.14 |  | |  |  |
| S(BW) | | 1 | 1784.01 |  | |  |  |
| S(C) | | 2 | 1176.70 |  | |  |  |
| T × L | | 12 | 892.93 |  | |  |  |
| T × BW vs C | | 6 | 1498.60 | 5.74 | | 0.01 | T × S(C) |
| T × Between C | | 6 | 287.25 | 1.10 | | 0.42 | T × S(C) |
| T × S(L) | | 18 | 460.11 |  | |  |  |
| T × S(BW) | | 6 | 857.74 | 3.28 | | 0.04 | T × S(C) |
| T × S(C) | | 12 | 261.29 | 1.65 | | 0.08 | Res |
| Residual | | 294 | 157.98 |  | |  |  |

Appendix S3 cont.

| **Predators**, Cochran’s test, C = 0.38, P < 0.01 | | | |  |  |
| --- | --- | --- | --- | --- | --- |
| Source of variation | df | MS | F | P | F vs. |
| Time, T | 6 | 27.48 |  |  |  |
| Locations, L | 2 | 0.44 |  |  |  |
| BW vs C | 1 | 0.01 | 0.01 | 0.95 | Bet C |
| Between C | 1 | 0.88 | 0.01 | 0.92 | T × Bet C |
| Site(L), S(L) | 3 | 15.39 |  |  |  |
| S(BW) | 1 | 2.29 | 13.02 | 0.00 | T × S(C)/ S(BW) |
| S(C) | 2 | 21.95 | 0.74 | 0.50 | T × S(C) |
| T × L | 12 | 55.99 |  |  |  |
| T × BW vs C | 6 | 36.60 | 0.49 | 0.80 | T × Bet C |
| T × Between C | 6 | 75.38 | 2.53 | 0.08 | T × S(C) |
| T × S(L) | 18 | 23.01 |  |  |  |
| T × S(BW) | 6 | 9.54 | 3.12 | 0.04 | T × S(C)/ T × S(BW) |
| T × S(C) | 12 | 29.75 | 1.31 | 0.21 | Res |
| Residual | 294 | 22.67 |  |  |  |
|  |  |  |  |  |  |
| **Predators excluding large schools**, Cochran’s test, C = 0.22, P < 0.01 | | | | |  |
| Source of variation | df | MS | F | P | F vs. |
| Time, T | 6 | 1.76 |  |  |  |
| Locations, L | 2 | 25.39 |  |  |  |
| BW vs C | 1 | 48.21 |  |  |  |
| Between C | 1 | 2.57 |  |  |  |
| Site(L), S(L) | 3 | 1.45 |  |  |  |
| S(BW) | 1 | 2.29 |  |  |  |
| S(C) | 2 | 1.04 |  |  |  |
| T × L | 12 | 5.59 |  |  |  |
| T × BW vs C | 6 | 9.17 | 4.57 | 0.04 | T × Bet C |
| T × Between C | 6 | 2.01 | 1.97 | 0.07 | Res |
| T × S(L) | 18 | 3.93 |  |  |  |
| T × S(BW) | 6 | 9.54 | 8.44 | 0.00 | T × S(C) |
| T × S(C) | 12 | 1.13 | 1.11 | 0.35 | Res |
| Residual | 294 | 1.02 |  |  |  |

**Appendix S4**: Analyses of variance testing the difference between six measures of the fish assemblage between areas of the seawall with or without flowerpots at Blackwattle Bay at ten times of sampling after pot deployment (n = 4). Treatment (flowerpot vs control) was fixed and orthogonal with 2 levels, Time was a random factor with 10 levels and Site was a random factor with 2 levels.

| **Species density**, Cochran’s test, C = 0.06, P > 0.05 | | | |
| --- | --- | --- | --- |
| Source of variation | df | MS | F |
| Time, T | 1 | 4.51 | 1.25 |
| Site, S | 9 | 1.06 | 0.29 |
| Treatment, Tr | 1 | 7.66 | no test |
| T × S | 9 | 3.63 | 5.56*** |
| Tr × T | 1 | 2.56 | 2.51 |
| Tr × S | 9 | 0.51 | 0.50 |
| Tr × T × S | 9 | 1.02 | 1.56 |
| Res | 120 | 0.65 |  |
|  | | | |
| **Herbivores**, Cochran’s test, C = 0.33, P < 0.01 | | | |
| Source of variation | df | MS | F |
| Time, T | 1 | 5.41 | 1.42 |
| Site, S | 9 | 6.40 | 1.68 |
| Treatment, Tr | 1 | 0.00 | 0.00 |
| T × S | 9 | 3.80 | 5.26*** |
| Tr × T^p^ | 1 |  |  |
| Tr × S^p^ | 9 |  |  |
| Tr × T × S^p^ | 9 |  |  |
| Res | 120 | 0.65 |  |
| 1-pooled | 129 | 0.75 |  |
| 2-pooled | 130 | 0.75 |  |
| 3-pooled | 139 | 0.72 |  |
|  |  |  |  |
| **Omnivores**, Cochran’s test, C = 0.42, P < 0.01 | | | |
| Source of variation | df | MS | F |
| Time, T | 1 | 0.22 | 1.07 |
| Site, S | 9 | 0.03 | 0.12 |
| Treatment, Tr | 1 | 0.90 | 0.00 |
| T × S | 9 | 0.21 | 3.79 |
| Tr × T | 1 | 0.22 | 1.07 |
| Tr × S | 9 | 0.03 | 0.12 |
| Tr × T × S | 9 | 0.21 | 0.21*** |
| Res | 120 | 0.05 |  |
| Appendix S4 cont. |  |  |  |
| **Planktivores**, Cochran’s test, C = 0.42, P < 0.01 | | | |
| Source of variation | df | MS | F |
| Time, T | 1 | 1915.00 | 2.23 |
| Site, S | 9 | 3036.31 | 3.53 |
| Treatment, Tr | 1 | 5.26 | no test |
| T × S | 9 | 860.03 | 2.63** |
| Tr × T | 1 | 356.01 | 0.72 |
| Tr × S | 9 | 88.51 | 0.18 |
| Tr × T × S | 9 | 492.73 | 1.51 |
| Res | 120 | 326.61 |  |
|  |  |  |  |
| **Predators**, Cochran’s test, C = 0.53, P < 0.01 | | | |
| Source of variation | df | MS | F |
| Time, T | 1 | 50.11 | 2.58 |
| Site, S | 9 | 9.51 | 0.49 |
| Treatment, Tr | 1 | 1.81 | no test |
| T × S | 9 | 19.40 | 2.28 |
| Tr × T | 1 | 2.97 | 0.13 |
| Tr × S | 9 | 54.06 | 2.34 |
| Tr × T × S | 9 | 23.14 | 2.72*** |
| Res | 120 | 8.51 |  |
|  |  |  |  |
| **Predators excluding large schools**, Cochran’s test,  C = 0.20, P < 0.01 | | | |
| Source of variation | df | MS | F |
| Time, T | 1 | 6.41 | 0.55 |
| Site, S | 9 | 2.50 | 0.22 |
| Treatment, Tr | 1 | 0.90 | no test |
| T × S | 9 | 11.59 | 6.90*** |
| Tr × T | 1 | 3.25 | 1.93 |
| Tr × S | 9 | 4.23 | 2.51 |
| Tr × T × S^p^ | 9 |  |  |
| Res | 120 |  |  |
| 1-pooled | 129 |  |  |
| *P < 0.05, **P < 0.01, ***P < 0.001 | | |  |


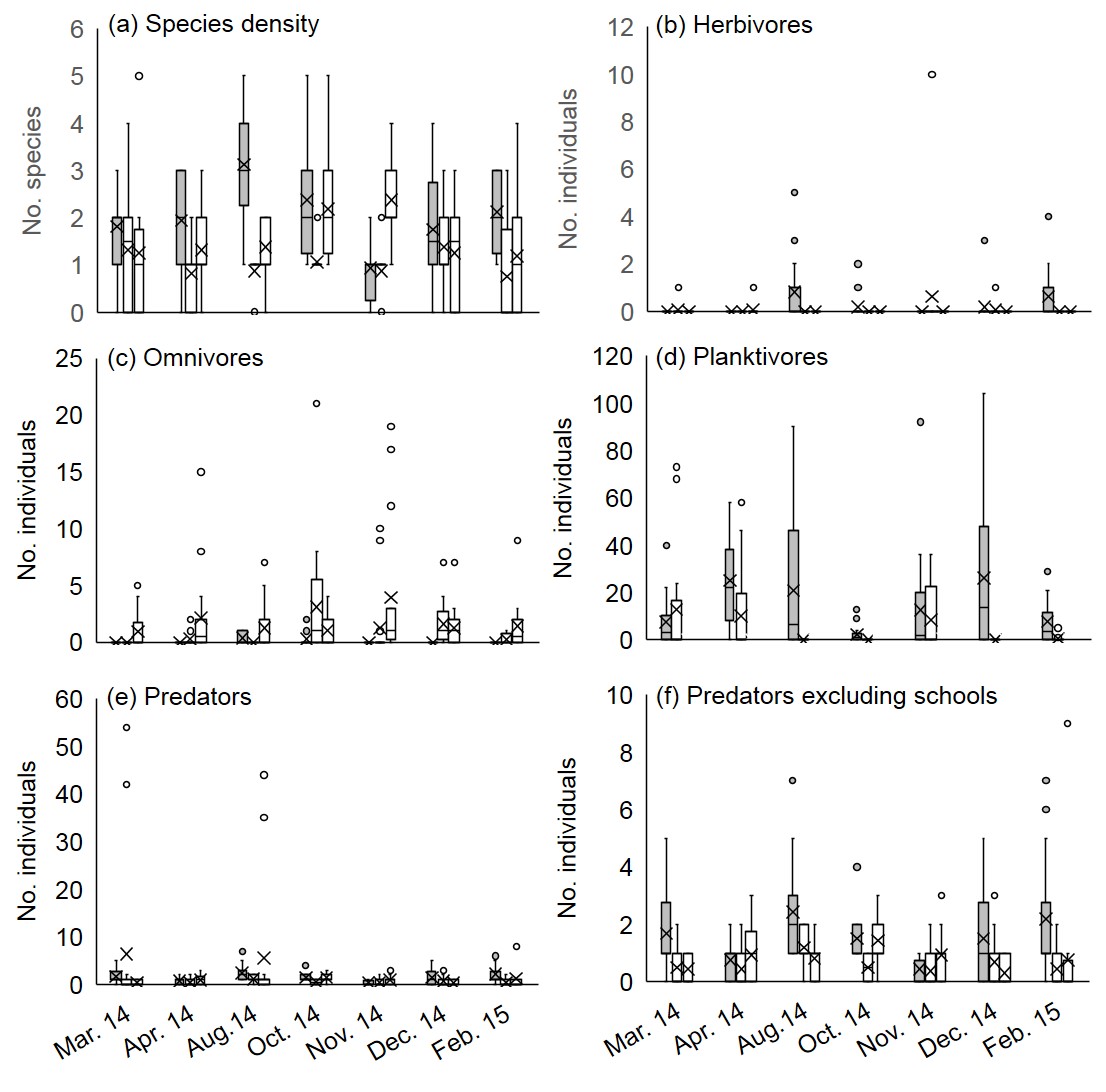


**Appendix S5.** a) Species density and b-f) relative abundance of fish at Blackwattle Bay (with flowerpots, grey bars) and two control locations (white bars) over 7 sampling times (n = 8). Upper and lower hinges: first and third quartiles; mid-line: median; cross: mean; whiskers: points within 1.5 × interquartile range; dots represent data outside of 1.5 × interquartile range.


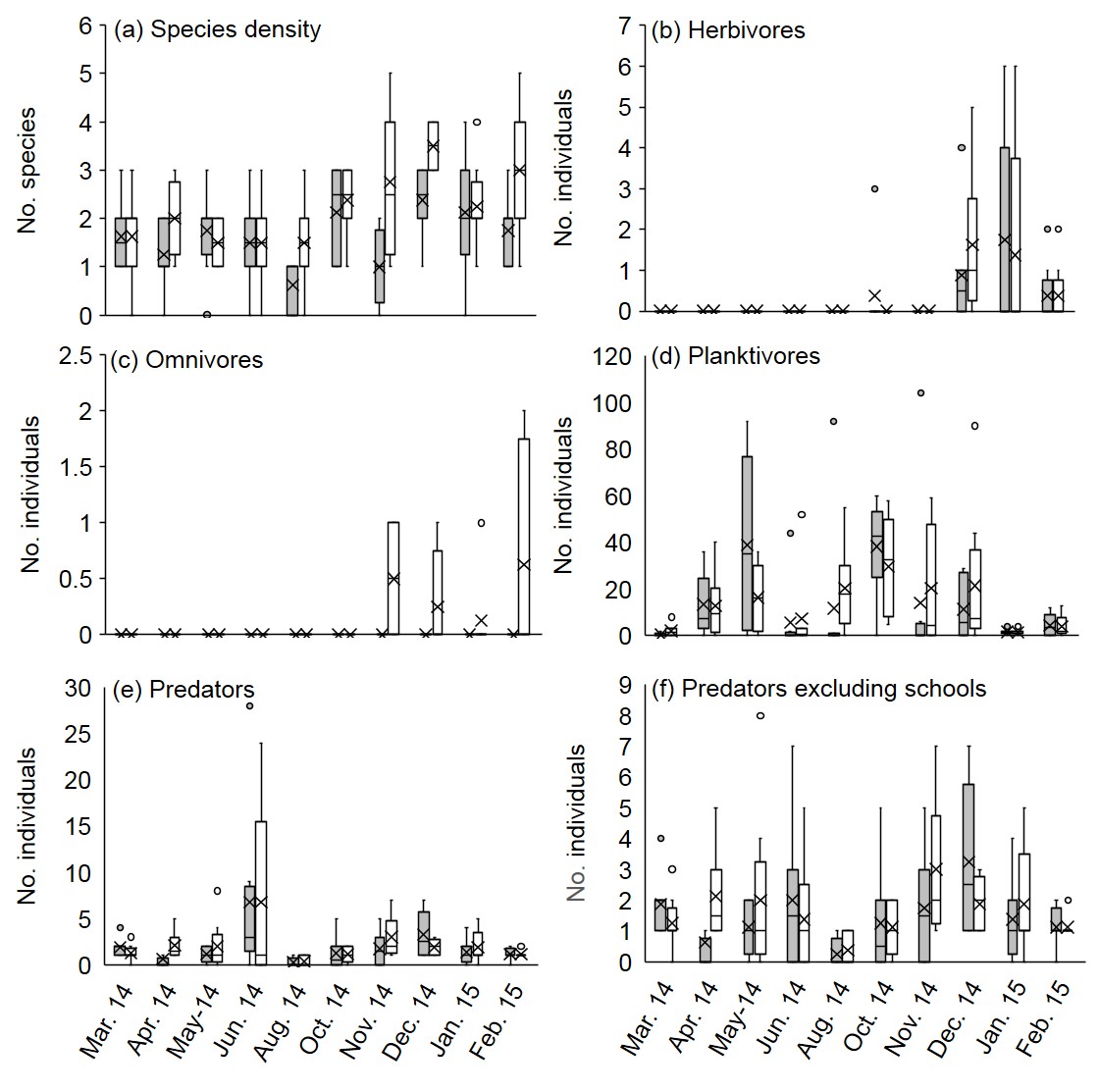


**Appendix S6.** a) Species density and b-f) relative abundance of fish at areas of the seawall with (grey bars) or without (white bars) flowerpots over 10 sampling times (n = 4). Upper and lower hinges: first and third quartiles; mid-line: median; cross: mean; whiskers: points within 1.5 × interquartile range; dots represent data outside of 1.5 × interquartile range.
